# Evidence of Repeated and Independent Saltational Evolution in a Peculiar Genus of Sphinx Moths (*Proserpinus*: Sphingidae)

**DOI:** 10.1371/journal.pone.0004035

**Published:** 2008-12-24

**Authors:** Daniel Rubinoff, Johannes J. Le Roux

**Affiliations:** Department of Plant and Environmental Protection Sciences, University of Hawaii at Manoa, Honolulu, Hawaii, United States of America; University of Sheffield, United Kingdom

## Abstract

**Background:**

Saltational evolution in which a particular lineage undergoes relatively rapid, significant, and unparalleled change as compared with its closest relatives is rarely invoked as an alternative model to the dominant paradigm of gradualistic evolution. Identifying saltational events is an important first-step in assessing the importance of this discontinuous model in generating evolutionary novelty. We offer evidence for three independent instances of saltational evolution in a charismatic moth genus with only eight species.

**Methodology/Principal Findings:**

Maximum parsimony, maximum likelihood and Bayesian search criteria offered congruent, well supported phylogenies based on 1,965 base pairs of DNA sequence using the mitochondrial gene *cytochrome oxidase subunit I*, and the nuclear genes *elongation factor-1 alpha* and *wingless*. Using a comparative methods approach, we examined three taxa exhibiting novelty in the form of Batesian mimicry, host plant shift, and dramatic physiological differences in light of the phylogenetic data. All three traits appear to have evolved relatively rapidly and independently in three different species of *Proserpinus*. Each saltational species exhibits a markedly different and discrete example of discontinuous trait evolution while remaining canalized for other typical traits shared by the rest of the genus. All three saltational taxa show insignificantly different levels of overall genetic change as compared with their congeners, implying that their divergence is targeted to particular traits and not genome-wide.

**Conclusions/Significance:**

Such rapid evolution of novel traits in individual species suggests that the pace of evolution can be quick, dramatic, and isolated—even on the species level. These results may be applicable to other groups in which specific taxa have generated pronounced evolutionary novelty. Genetic mechanisms and methods for assessing such relatively rapid changes are postulated.

## Introduction

Saltational evolution-rapid evolutionary change in a taxon while sister taxa remain relatively unchanged for the same suite of characters over equal or greater periods of time [1]- provides an important opportunity for understanding the reasons and rate at which selection (and/or drift) can foster evolutionary novelty in particular lineages. The concept of saltation, however, is difficult to reconcile with the dominant and contrasting theory of gradualistic change (Synthetic Theory [2], [3]), which rejects the idea of a dramatic rate discontinuity between sister taxa. Although saltational evolution is necessarily a qualitative macroevolutionary concept, irrespective of timescale, there are specific features that are indicative of the phenomenon. Saltational evolution does not imply genome-wide changes, as this might suggest that chance, in concert with an overall rapid rate of change, and not selection (in the typical Darwinian sense), are producing marked evolutionary differences simply as a function of a pervasive increase in the pace of evolution throughout a clade [4]. Rather, saltational evolution as we consider it here, is targeted and relatively rapid change in specific characters (and their coding genes) of apparent survival importance while the overall genome apparently evolves at the same pace as in non-saltational, comparatively static, sister taxa.

In order to demonstrate *in situ* saltational evolution it is thus essential to establish phylogenetic relationships between the purportedly saltational taxon and its sister taxon. It is expected for taxa exhibiting saltational character evolution to be nested within a group of taxa that almost universally retain ancestral states for those same traits, otherwise the apparently saltational event may have occurred much earlier in a deeper clade (and thus not quickly). The pace of change is not defined by empirical measurements of time (e.g. molecular clocks) but rather by relative levels of change in specific characters as compared with sister taxa, operating from a similar overall genome. Furthermore, the saltational taxon must show equal or smaller amounts of overall genomic divergence from the most recent common ancestor than do the non-saltational taxa in the same clade, otherwise the taxon in question may be an example of generally accelerated evolution rather than targeted, rapid change in a specific trait or suite of traits. Only this paradigm would clearly demonstrate that the saltational taxon has derived, *de novo*, a series of traits while the rest of the clade has remained in relative stasis. The suite of characters suggesting saltational evolution might vary, but should tend to be non-trivial and have apparent evolutionary significance and adaptive value.

For example, whales might not represent saltational evolution as we consider it here, because, despite their extreme divergence their sister taxa have also diverged from a common ancestor over a similarly long period of time. This is represented by large amounts of genome-wide genetic divergence between whales and artiodactyls [5], [6] and an extensive and gradual fossil record [7], [8]). On the other hand, if there were relatively close sister taxa for whales (e.g. terrestrial carnivores) that were genetically no more distant from whales than other terrestrial carnivores, then saltational evolution might be invoked. Under the current scenario whales represent dramatic evolutionary change but along relatively long branches (branch length changes) with intermediate forms.

**Figure 1:**
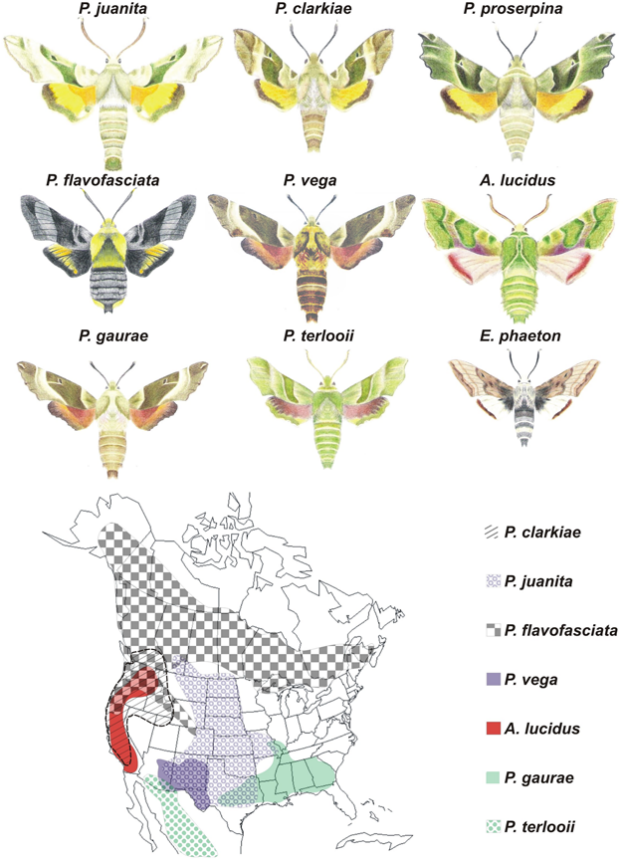
Illustrations of all species of *Proserpinus*, *Arctonotus* and *Euproserpinus* included in phylogenetic reconstructions in this study. The inserted map shows the distribution ranges of North American Proserpine species (excluding the outgroup genus *Euproserpinus* and the Paleoarctic *P. proserpina*).

The charismatic sphinx moth genera *Proserpinus* and its close relative *Arctonotus* [9] contain seven and one species respectively (Fig. 1.)- relatively small number(s), especially for insect genera. Although their large size and bright coloration make them a focus of attention with collectors most of the species appear to be very localized in their distribution and are very rarely collected, even though several are day flying and conspicuous [10]. Their biogeography is also remarkable with both *Arctonotus* and six of the seven *Proserpinus* species being restricted to subsections of North America while the single Paleoarctic species, *P. proserpina* occurs from western China and Siberia west to the Iberian peninsula and North Africa, a far larger range than any other species in the group (Fig. 1). The external morphology, host plant use, and phenology of *Proserpinus* and *Arctonotus* are remarkably canalized across most species. The adult moths are green with orange or red hind wings and fly very quickly during the day or twilight periods (but not night), hovering like miniature hummingbirds to take nectar from flowers. Depending on species and latitude adults are present for only a few weeks a year in the spring or early summer (or during the mid-summer monsoon in the desert southwest of North America). The larvae feed only on plants such as evening primrose and its relatives in the family Onagraceae.

There are, however, a few exceptions to this otherwise strict paradigm of behavioral and morphological traits which initially attracted our interest. One species, *P. flavofasciata*, has completely abandoned the green and red pattern and is an excellent black and yellow mimic of bumblebees (*Bombus* spp.) which share its boreal forest habitat; it is the only species that is not at all green (Fig. 1). Another species, *P. terlooii*, feeds only on ground spiderling (*Boerhaavia*) species in the family Nyctaginaceae, unrelated to Onagraceae and common during the monsoon in the upland desert habitat where the moth resides. It is the only species that does not feed on a plant in the family Onagraceae. Finally, the monotypic genus *Arctonotus* was made for the single species, *A. lucidus*, which is known to be a close relative of *Proserpinus* [9], [10]. *Arctonotus* differs in the form of a few morphological characters in the genitalia, structure of leg spines, and its robust densely-scaled body [11] but it is its life history that significantly sets it apart from *Proserpinus*, and in some respects, the rest of the hundreds of species in the subfamily Macroglossinae [9]. Adult *A. lucidus* are active in the early evening hours in mid winter along the Pacific Coast region of North America. Unlike *Proserpinus* whose members require both heat and moisture to emerge from pupation from March through August, *A. lucidus* emerges during the cold winter rains between November (Southern and Central California, U.S.) and April (Washington state, U.S. and British Columbia, Canada) when temperatures can regularly fall below 0°C. During this season there are virtually no blooming plants and perhaps as a result, *A. lucidus* adults do not feed. In fact, the moths cannot feed since their proboscis does not fully extend and is non-functional [9], [12]. Not only are the adult moths able to withstand unusually cold temperatures for an active adult insect but eggs and larvae continue to develop during the winter and are exposed to similar or even colder conditions during much of their development [12]. Such winter activity and freezing resistance or tolerance reveals significant physiological adaptations in both the adult and larval stages of *Arctonotus*. Furthermore, non-feeding and subsequent loss of functioning mouthparts in the adults requires the adult insect to rely wholly on energy acquired during its larval stage more than six months prior. Kawahara [9] has demonstrated that such a loss of adult feeding in an individual species is extremely rare in the family Sphingidae and therefore of particular significance.

The physiological, behavioral, and morphological changes evident in *Arctonotus* make it easily distinguishable from any *Proserpinus* and in combination with the unique changes in *P. flavofasciata* and *P. terlooii* inspired this study to investigate the evolutionary relationships between and within the two genera as they pertain to the pace and evolution of novel and unique traits. Specifically we sought to understand the evolution of *Arctonotus* and *Proserpinus* by answering the following questions: 1) What is the systematic relationship between *Arctonotus* and *Proserpinus*, and between species within *Proserpinus*?, 2) Is the propensity for novel and unique traits clustered in taxa from basal lineages, a more derived crown group, or neither? (such patterns might suggest that the divergent taxa are monophyletic with a derived propensity for differentiation or, if the divergent taxa are all from basal lineages, that they are retaining ancestral traits and not evolving new ones), and 3) Can systematic relationships and genetic distances in *Proserpinus* tell us something about the pace of evolution in the group and the possibility of saltational evolution in some taxa? A molecular phylogeny provides an ideal framework for establishing evolutionary relationships between the species in this peculiar group of moths and, by comparing the relative rates of genetic change from a common ancestor for the typical and divergent taxa, we can evaluate the evidence for saltational evolution in the genus.

## Results

### Proserpinus Systematics

**Figure 2:**
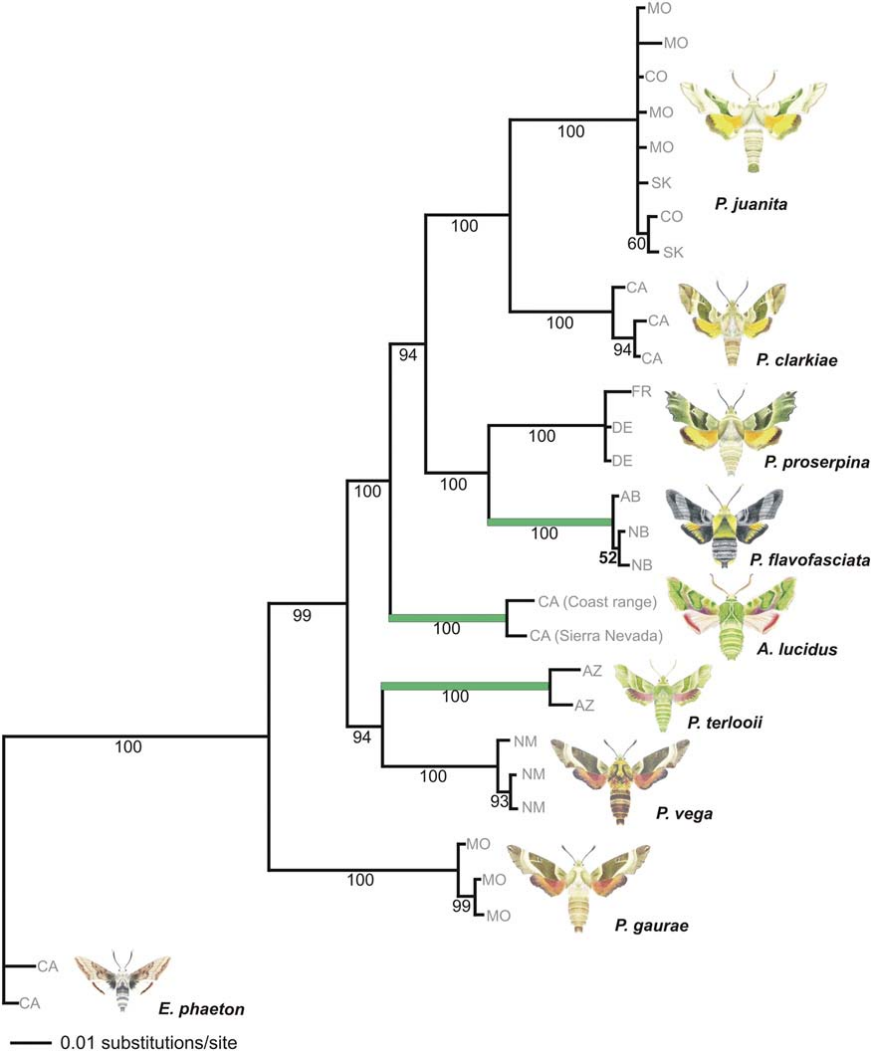
Bayesian consensus tree generated with posterior probabilities inferred from 1,965 bp of combined COI, EF1α, and wingless DNA sequence data. Branch support is given as posterior probabilities (numbers beneath branches). Accession location data are mapped onto the tree (MO, Missouri; CO, Colorado; AB, Alberta [Canada]; NB, New Brunswick [Canada]; SK, Saskatchewan [Canada]; FR, France; DE, Germany; CA, California; AZ, Arizona; NM, New Mexico). Saltational taxa are highlighted in green.

**Figure 3:**
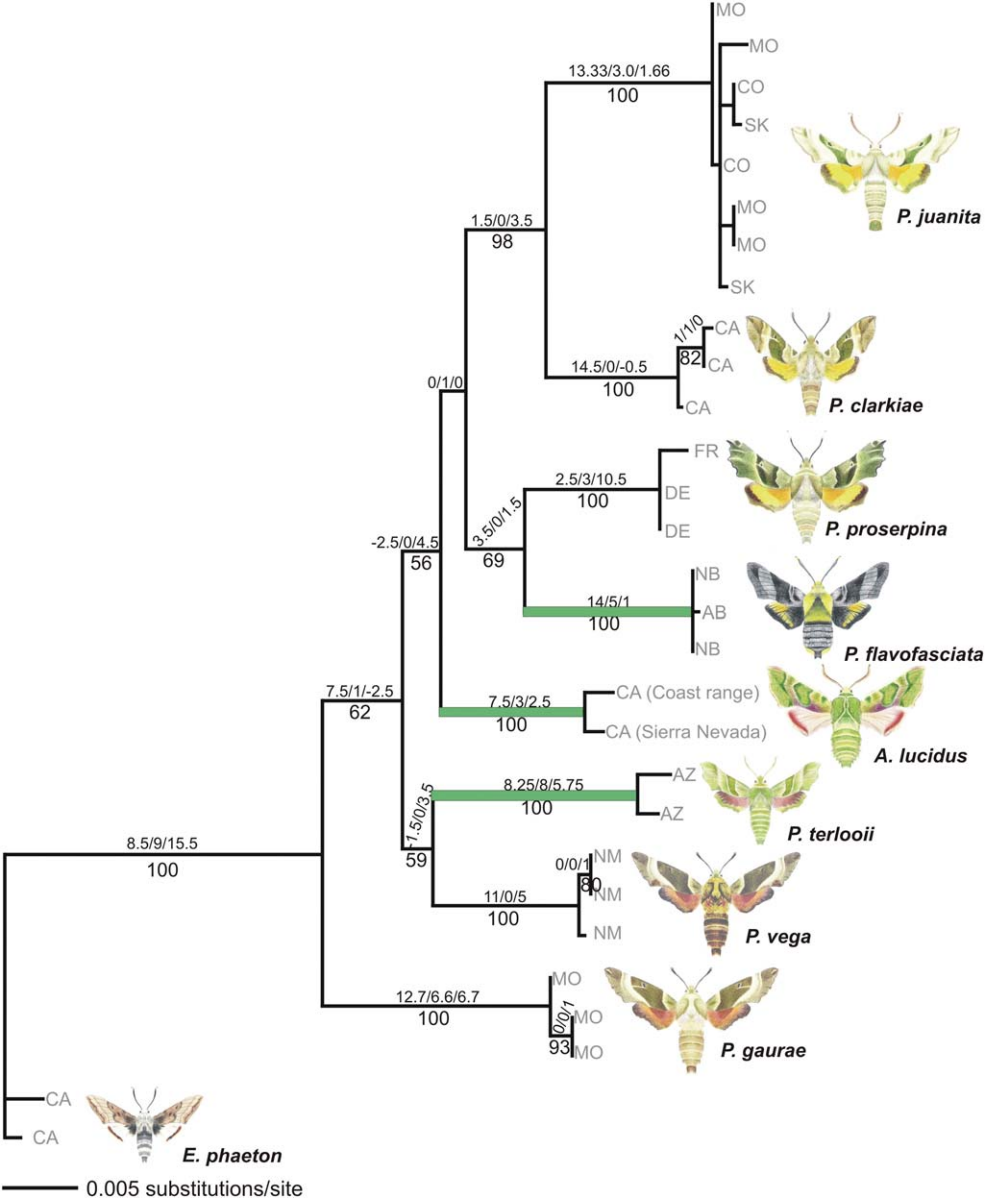
ML phylogram (−ln likelihood = 899.956) generated from the combined COI, EF1α, and wingless dataset using the GTR+I+G model of evolution. Branch support is given beneath branches as bootstrap values (1000 replicates). Numbers above branches indicate decay indices (MP) determined separately for COI, wingless and EF1 α, respectively. Accession location data are mapped onto the tree (MO, Missouri; CO, Colorado; CAN, AB, Alberta [Canada]; NB, New Brunswick [Canada]; SK, Saskatchewan [Canada]; FR, France; DE, Germany; CA, California; AZ, Arizona; NM, New Mexico). Saltational taxa are highlighted in green.

The best-fit maximum likelihood (ML) model was a general time reversible model plus an estimated proportion of invariable sites and heterogeneity amongst rates [GTR+I+G, with *R* matrix (2.3419, 9.3002, 10.0393, 1.8624, 33.0687, 1.000); base frequencies (*A* = 0.2711, *C* = 0.2324, *G* = 0.2174, *T* = 0.2790); shape of gamma-distribution 0.6365; and proportion of invariable sites 0.6246] and the score of the optimal tree was −ln likelihood = 899.956. Bayesian posterior probability estimates from MrBayes yielded 200,002 trees of which 196,002 were sampled. Maximum parsimony (MP) analysis yielded 18,300 most parsimonious trees with 308 steps. Bayesian analysis yielded the exact same topology as the ML analysis (Figs 2, 3). Maximum parsimony (MP), maximum likelihood (ML) and Bayesian posterior probability estimates all converged on the same topology for the combined dataset analysis. The only topological difference was slightly poorer resolution regarding the relative placement of *Arctonotus* and the *P. proserpina/flavofasciata* clade for the MP topology (results not shown).

Branch support was high for almost all nodes for all three search criteria, though generally higher for Bayesian and MP than ML analyses (Figs 2 and 3). While the support for *Proserpinus/Arctonotus* as a monophyletic with respect to *Euproserpinus* was unequivocal, all phylogenies rendered *Proserpinus* paraphyletic with respect to *Arctonotus* (Figs 2 and 3). Paraphyly was strongly supported by all three search criteria. In all three search criteria *P. terlooii/vega*, *proserpina/flavofasciata*, and *clarkiae/juanita* formed well-supported monophyletic sister species relationships. *Proserpinus gaurae* is the most basal branching lineage, followed by a branch that leads to the *P. terlooii/vega* clade. *Arctonotus lucidus* branches next in both Bayesian and ML topologies, but MP has *Arctonotus* unresolved in a polytomy with the *P. flavofasciata/proserpina* and *clarkiae/juanita* clades (results not shown). In contrast, both the Bayesian and ML phylogenies have the *P. flavofasciata/proserpina* and *clarkiae/juanita* clades branched apically, after *Arctonotus*. These results strongly suggest that all three divergent taxa that have evolved unique traits (*A. lucidus*, *P. terlooii*, *P. flavofasciata*), are neither monophyletic, basal, nor particularly derived in our phylogenetic reconstructions. Further, the dramatic differences among these three taxa, which lead to a separate generic designation for *Arctonotus*, appear to have arisen relatively quickly and independently within *Proserpinus*. *Arctonotus lucidus* is, in fact, another species of *Proserpinus*. A systematic revision of the genera will be the subject of another paper.

### Saltational Evolution

Relative rate tests comparing DNA sequence substitution rates indicated that no significant differences exist in the rate of DNA sequence evolution among putative saltational and non-saltational sister taxa (*P* values ranging from 0.26–0.74). This is an essential factor in distinguishing saltational evolutional from general accelerated evolution. Accelerated evolution is remarkable because it indicates that a particular species or clade is undergoing an increased rate of change across the board at the genomic level [e.g. 13]. In contrast saltation assumes a similar pace of overall genetic evolution but rapid evolution of novel phenotypic, physiological, or behavioral features in one or few members of a monophyletic clade. *Proserpinus terlooii*, *P. flavofasciata* and *Arctonotus lucidus* are all centrally located in the genus phylogeny and have rates of genetic change comparable to the rest of their congeners but rapid pronounced evolution (saltation) in specific functional character suites (host plant use, mimicry, cold weather adaptation). Saltational evolution suggests that an organism need not have an accelerated overall mutational rate to rapidly acquire functionally important autapomorphic traits. In the broader context of evolution these results suggest that organisms do not require broadly accelerated rates of genomic change to undergo rapid and dramatic adaptations.

## Discussion

While the evolution of mimicry, novel host plant use, or dramatic physiological changes are not, in a broader context, unique, the pace at which they have arisen and their apparent saltational origin in *Proserpinus* is remarkable. Even more unusual is that each divergence from the canalized pattern has been in a different subclade within this small genus, suggesting that the saltational breaks have been independent rather than the expression of a propensity in a particular subclade. Timing the divergences of the saltational taxa without a fossil record is, at best, imperfect. But employing Brower's [14] broad estimate of 2% mtDNA divergence per million years, as estimated for butterflies, suggests that *P. flavifasciata* diverged 5.5, *A. lucidus*, 4.8 and *P. terlooii* 4.6 million years ago from their respective sister taxa, implying that the timing of these events may not be correlated. To our knowledge, this is the first report of such a phenomenon (multiple rapid saltational events within a single small genus).

Saltational evolution is as much about the stasis in sister taxa as it is about the derivation of novelty in those taxa experiencing rapid change since saltation refers to an isolated acceleration in evolutionary change of specific traits. Therefore, while many different species might evolve novel host plant use, mimicry, or novel physiology, these changes would not be saltational in a clade whose members are all actively evolving related, novel features over roughly equal periods of time. Because all but one species of *Proserpinus* are canalized for host plant use, or color pattern, or phenology and physiology, the rapid derivation of divergent unique traits, each in a separate species, strongly suggest that these adaptations are saltational.

### Biogeography and Mimicry

There has been much discussion regarding the importance of biogeography in the evolution of Mullerian mimicry and its pace in tropical American butterflies (e.g. [14], [15], [16], [17]. In contrast, the pace and mechanisms of evolution of Batesian mimicry in Lepidoptera, when it can be established clearly (e.g. [18]) is poorly known [19]. The phylogeography of *Proserpinus* suggests a relatively short period of time for the evolution of mimicry in *P. flavofasciata*. Because *P. proserpina* is the only member of *Proserpinus*, *Arctonotus* and *Euproserpinus* that occurs in the Old World and is not a basal taxon, it is parsimonious to assume a New World origin for the three genera. It is plausible that during an interglacial period the common ancestor of *P. flavofasciata* and *P. proserpina* (strongly supported sister taxa) invaded northern regions of North America (to which *P. flavofasciata* is currently restricted), and the ancestor of *P. proserpina* then spread across Beringia to Asia and Europe or from Canada into Europe directly. Under either dispersal scenario, only after the typical green and orange *P. proserpina* diverged did *P. flavofasciata* undergo a complete change in color pattern and become a bumble bee mimic. During this same period of time, as before, no other *Proserpinus* (now including *A. lucidus*) diverged from the green forewing pattern, despite the fact that all *Proserpinus* are fully sympatric with species of *Bombus*.

Perhaps gradual selection did not favor mimicry in *Proserpinus* because lowered fitness of intermediate phenotypes (akin to a valley on the adaptive landscape [20], [21] limited selection in that direction; although there may be some survival benefit for poor mimics, rapid, drastic change (saltation) to a close mimic is more likely [19]. A saltational event allowed a bumble bee mimic to cross the fitness “valley” and reach an alternative fitness “peak” or at least slope thereof [20], [21]. Indeed, concerted evolution in basal *Proserpinus* species appears to maintain the ancestral coloration (green and red) despite a wide environmental range (climate and latitude). Such saltational change to mimicry in body coloration might usually lead to too much change to confer a selective advantage and lead to rapid extinction. But in the case of *P. flavofasciata* such change(s) rendered higher fitness through Batesian mimicry and this saltational species has benefited. Further investigation of the mechanisms behind this unique and rapid evolution of a bumblebee phenotype will be important in understanding the evolution of mimicry.

### Host Plant Shift

It is common for groups of phytophagous insects to harbor both generalists and specialists, with specialists generally outnumbering generalists [22]. Perhaps as a result, host plant use in many phytophagous insect lineages is taxonomically conserved [22] with monophyletic insect lineages usually feeding on closely related plants. Even in this context *Proserpinus* is extremely conservative with all but one species feeding on a few genera within a single plant family Onagraceae and nothing else. Further, there is evidence that many host plant shifts in Lepidoptera are in fact reversions in that some insect species may have a propensity to use host plants still used by closely related taxa through “evolutionary memory” of ancestral use [22]. The unique host plant shift in *P. terlooi* does not appear to follow the paradigm of ancestral reversion and defies the extremely conservative nature of most Proserpine host plant use. *Proserpinus terlooii* has shifted onto, and only feeds upon, two species in the genus *Boerhaavia* (Nyctaginaceae) notable not only because of the phylogenetic distance between the host plants involved in this shift, but also the pace of this change. It stands in sharp contrast to the strict specialization across all other species in the two genera (including the outgroup genus *Euproserpinus*) which are solely found on Onagraceae (and where sympatric, these taxa can be found on the same host plants). The importance of context in considering saltation is perhaps best illustrated by what might appear to be this least unusual saltational phenomenon; the host family switch to Nyctaginaceae in *P. terlooii*. For insects in general host plant shifts across species are well-known and generalist species may feed on multiple families of plants. But *P. terlooii* must be considered in the evolutionary context of *Proserpinus*, *Arctonotus*, and *Euproserpinus*, three genera all of whose species, except *P. terlooii*, are solely restricted to Onagraceae. Therefore, it is not simply that *P. terlooii* has made a host plant family shift, it is that *P. terlooi* is the *only* species to have made *any* host shift across the three genera. Saltation appears to be supported as all phylogenies demonstrate that this host plant shift was likely rapid and unique among *Proserpinus*, including *P. terlooii*'s sister taxon, *P. vega*, with which *P. terlooii* is sympatric over large portions of its range (Fig. 1). There is evidence that host shifts like the proposed saltational event observed in *Proserpinus* may convey a survival advantage through the acquisition of enemy-free space [23], [24].

### Physiology and Phenology

Nectar is an important source of energy and nutrients in most adult Lepidoptera [25] and adult fecundity significantly improves through nutrient acquisition. Although some Lepidoptera do not feed as adults, shifts to a non-feeding life phase require significant physiological, developmental, and behavioral changes since all resources for adult development, activity, and reproduction (including all egg/sperm generation) must be acquired and stored during the larval stage. Abandoning the ability to feed as an adult is therefore a significant evolutionary change. This significance is also supported by the rarity with which non-feeding has evolved since it appears to be evolutionarily canalized and phylogenetically conserved in the Sphingidae [9]. *Arctonotus lucidus* is the only member of the subfamily Macroglossinae in which adults do not feed. Whether non-feeding occurred in concert with cold-tolerance physiological changes allowing the species to go through most of its active lifecycle in freezing or near–freezing temperatures remains unclear. However, the saltational nature of these dramatic shifts is revealed by *Arctonotus*' central placement and comparative low level of genetic divergence within *Proserpinus*.

In all three circumstances of saltational evolution in *Proserpinus*, there are sister taxa confronted by apparently similar selection pressures in similar environments. For example, *P. flavofasciata* is at least partially sympatric with *P. clarkiae* (Fig. 1) and occurs at similar latitudes to its sister species *P. proserpina* (which does not inhabit boreal forest, but ranges up to boreal forest in Europe and Asia). Similarly, *P. terlooii* shares much of its geographic range with its sister species, *P. vega*, though the latter appears to occur at higher altitudes. *Arctonotus* is almost completely sympatric with *P. clarkiae* (Fig. 1) and/or *Euproserpinus* species, yet it is the only cold-weather adapted, non-feeding species. These situations suggest that it is not unique selection pressures such as access to a habitat or host plant, or climate that fostered evolutionary novelty but rather saltation–rapid and dramatic character-based changes-that led to a unique response(s) to said condition(s) by particular species. This may be an important aspect of the evolutionary process, at least in *Proserpinus*.

### Why Saltational Evolution?

While the discontinuous patterns we demonstrate in *Proserpinus* are noteworthy, some might suggest that there may be other equally discontinuous patterns of character evolution between species in other groups. We quite agree. One of our goals with this data has been to demonstrate a relatively simple standard by which evidence for saltational evolution might be demonstrated phylogenetically in a variety of groups. We suspect that saltation may be a more common occurrence than is currently appreciated. For example, unique characters such as novel detoxification proteins (e.g. p450s) for plant compounds in phytophagous insects might rightly be considered saltational if they occur in a phylogenetic context that lacks such novelty. The alternative to the analysis we propose is a potentially counterproductive (and we would argue arbitrary) standard for demonstrating saltation that is biased to ignore discontinuous evolution in favor of a gradualistic dogma. If we confine the idea of saltational evolution to only the most dramatic, and globally unprecedented changes between species, with no consideration of phylogenetic context for change, we may be ignoring a frequent, though less extreme, discontinuous process that is important in the generation of evolutionary novelty.

### Conclusions

Even though discontinuity is evident in the fossil record [26], saltationism appears to be hard to find in extant taxa. Given the likely fitness depression that might accompany any interbreeding with modal individuals, directional selection will act strongly against the spread of most saltations through populations, following their mutational origin in single individuals [1]. Despite these odds, saltationism has been implicated in a very few instances as an evolutionary force in some small wild populations of, for example, plants [27], [28] and insects [29], [30]. The consequences of saltationism are best described when considering Sewall Wright's [20], [21] adaptive landscape describing a taxon's fitness as a consequence of its genotypic composition (allele frequencies). Rare cases of successful saltation in nature represent displacement of a taxon on the adaptive landscape to an alternative fitness “peak” or slope thereof. Gradualistic evolution (Darwinian evolution, Synthetic Theory) will prevent selection towards such alternative fitness peaks as the lowered fitness associated with allele frequency changes and subsequent phenotypic changes in “valleys” between peaks would prevent directional evolution. Demonstrating the genetic basis for saltation through genome mapping methods, such as quantitative trait loci (e.g.[31]) and DNA microarray analysis may be a pathway to understanding how many genes are involved in each saltational trait. This is a daunting task but will be an important consideration for future research efforts aimed at understanding the underlying mechanisms of this phenomenon. Additionally, it may be found that saltational evolution is concentrated in certain taxonomic groups, while others do not display such a propensity. Such observations might suggest inherited suites of genes that promote rapid change without deterministic products (i.e. *Hox* genes). A number of homeotic and developmental regulatory genes have been characterized in insects (e.g. [32], [33]), and the given evidence for three independent cases of saltation reported here, may make *Proserpinus* an ideal model system for studying such processes.

## Materials and Methods

### Species Collection

We obtained multiple samples of all species in the genera *Proserpinus* and *Arctonotus*; fresh or dried tissue was placed directly in a −80°C freezer or stored in 100% EtOH until a freezer became available. Samples of two populations of *Euproserpinus phaeton* were included as a putative outgroup since the monophyly of *Euproserpinus*, *Arctonotus* and *Proserpinus* is well-established [9], [10]. Details concerning the taxa used in this study can be found in Table 1.

**Table 1:** Specimen data.

Taxon/Specimen ID	Location data*	GenBank Accessions§
*Proserpinus juanita*		
JLR24	Canada, Saskatchewan	FJ001498/FJ001527/FJ001556
JLR25	Canada, Saskatchewan	FJ001499/FJ001528/FJ001557
DR146	MO, St. Francois co.	FJ001475/FJ001504/FJ001533
DR147	MO, St. Francois co.	FJ001474/FJ001503/FJ001532
DR157	MO, St. Francois co.	FJ001490/FJ001519/FJ001548
DR158	MO, St. Francois co.	FJ001491/FJ001520/FJ001549
DR163	CO, Douglas co.	FJ001476/FJ001505/FJ001534
DR164	CO, Douglas co.	FJ001477/FJ001506/FJ001535
*Proserpinus clarkiae*		
DR141	CA, Amador co.	FJ001482/FJ001511/FJ001540
DR142	CA, El Dorado co.	FJ001493/FJ001522/FJ001551
DR152	CA, Amador co.	FJ001484/FJ001513/FJ001542
*Proserpinus proserpina*		
JLR28	Germany, Rheinland-Pfalz	FJ001502/FJ001531/FJ001560
DR143	France, Provence, Var	FJ001480/FJ001509/FJ001538
DR153	Germany, Rheinland-Pfalz	FJ001485/FJ001514/FJ001543
*Proserpinus flavofasciata*		
JLR20	Canada, Alberta	FJ001497/FJ001526/FJ001555
JLR27	Canada, New Brunswick	FJ001501/FJ001530/FJ001559
DR140	Canada, New Brunswick	FJ001481/FJ001510/FJ001539
*Proserpinus terlooii*		
DR144	AZ, Santa Cruz co.	FJ001479/FJ001508/FJ001537
DR155	AZ, Cochise co.	FJ001486/FJ001515/FJ001544
*Proserpinus vega*		
DR161	NM, Torrance co.	FJ001487/FJ001516/FJ001545
DR162	NM, Torrance co.	FJ001494/FJ001523/FJ001552
DR167	NM, Torrance co.	FJ001488/FJ001517/FJ001546
*Proserpinus gaurae*		
DR160	MO, St. Francois co.	FJ001489/FJ001518/FJ001547
DR185	MO, St. Francois co.	FJ001495/FJ001524/FJ001553
DR186	MO, St. Francois co.	FJ001496/FJ001525/FJ001554
*Arctonotus lucidus*		
DR145	CA, Solano co.	FJ001478/FJ001507/FJ001536
DR151	CA, Tuolumne co.	FJ001483/FJ001512/FJ001541
*Euproserpinus phaeton*		
JLR26	CA, Kern co.	FJ001500/FJ001529/FJ001558
DR174	CA, San Benito co.	FJ001492/FJ001521/FJ001550

* For all US specimen locations are given as state and county, all locations outside of the US are given as country and province.

§ Accession numbers for each specimen are given in the following order: CO1/EF1α/wingless.

### DNA Isolation, PCR Amplification and Sequencing

Total genomic DNA was extracted according to the manufacturer's protocol with the Qiagen DNA Tissue kit (Qiagen, USA) and stored at −80°C.

For all accessions we amplified three different gene regions; the mitochondrial gene cytochrome oxidase I (COI, 799 base pairs[bp]), and the nuclear genes elongation factor-1 α (EF1 α, 747 bp), and wingless (Wg, 419 bp). Because the mitochondrial and nuclear genomes are subject to different processes of inheritance, recombination, and selection it is important to use information from both genomes to comprehensively investigate evolutionary relationships [34], [35], [36]. We chose the three genes because they are functionally (and in the case of COI, genomically) independent and have all been demonstrated to be informative in distinguishing populations, species, or genera and therefore evolve at a pace that will be useful for answering the questions we present. Gene regions were PCR amplified and sequenced using the following primers: COI, Jerry (5′ CAA CAT TTA TTT TGA TTT TTT GG) and Pat (5′ ATC CAT TAC ATA TAA TCT GCC ATA); EF1 α, Oscar (5′ GGC CCA AGG AAA TGG GCA AGG G) and Bosie (5′ CCG GCG ACG TAA CCA CGA CGC); and Wg, LepWG1 (5′ GAR TGY AAR TGY CAY GGY ATG TCT GG) and LepWG2 (5′ ACT ICG CAR CAC CAR TGG AAT GTR CA). Each 50 µL reaction contained 18 µL HotMasterMix (HotMaster Taq DNA polymerase, 0.3 U; 2.5×HotMaster Taq Buffer pH 8.5, 45 mM KCl and 2.5 mM MgCl_2_; 200 µM of each dNTP; [Brinkman Instruments, Inc., USA]), 25 pmol of each primer and approximately 5 ng total genomic DNA. A thermocycle of 35 cycles: denaturation for 1 min at 94°C, locus-specific annealing (COI, 50°C; EF1 alpha, 55°C; Wg, 52°C) for 1 min, and extension for 1 min at 72°C was used for PCR amplification. All PCR products were purified with the QIAquick PCR purification kit (Qiagen, USA). Purified PCR products for all genes were sequenced in both directions by the University of Hawaii's Pacific Biosciences Research Center facility (http://core.biotech.hawaii.edu/) and were run on an ABI 377 automated sequencer (Applied Biosystems, USA.) using standard dye-terminator chemistry following the manufacturer's protocol. All DNA sequence data were deposited in GenBank (http://www.ncbi.nlm.nih.gov/).

### Phylogenetic Analysis

While it is common for different genes to suggest different systematic relationships due to a variety of factors including incomplete lineage sorting [37], combined analysis of all available data provides the most robust estimates of phylogenetic relationships [38]. Because most nuclear genes evolve at a much slower rate than mitochondrial regions like COI [39], many of the relationships supported by the nuclear genes may be poorly supported (simply due to a lack of data, rather than homoplasy) until analyzed in a combined dataset. For these reasons we chose to combine the data from the three genes into a single dataset for subsequent phylogenetic analysis.

The combined dataset was analyzed under maximum parsimony, maximum likelihood and Bayesian search criteria. Maximum parsimony analyses was done using PAUP* 4.0b10 with the option “Heuristic search” with TBR, MULTREES and COLLAPSE (max) options in effect [40]. Confidence in tree topologies was assessed using bootstrap analysis of 1000 replicates [41] as implemented in PAUP* 4.0b10 and partitioned Bremer support was calculated using TreeRot [42]. Maximum likelihood analysis was performed using parameter estimates obtained from the program MODELTEST version 3.06 [43]. Heuristic searches were carried out with TBR, MULTREES, and COLLAPSE options in effect; 1000 bootstrap replicates were used to assess branch support.

We used Mr. Bayes version 3.1 [44] to simultaneously estimate tree topology and optimize model parameters for the dataset. We implemented the standard 4 chain (one cold, three hot) search parameter in two simultaneous runs, with a burn-in value of 2000 trees (discarded before sampling) and a sample frequency of ten. Each chain was run for two million generations until the average standard deviation of split frequencies was 0.005 (well below the standard value of 0.01, implying that we could have searched for fewer generations and still been statistically confident that we were approaching an optimal solution), Trees were visualized in PAUP* 4.0b10 and TreeEdit Version 1.0a1-19 (http://evolve.zoo.ox.ac.uk/software/TreeEdit/main.html).

### Relative Rate Tests

Because the phenomenon of saltational evolution is ultimately scale-dependent, we tested for differences in DNA sequence evolution rates among putative saltational and non-saltational sister lineages (species) using the RRTree version 1.1 program [45]. Our reasoning behind using relative rate tests is that instances of saltational evolution would be supported if no significant differences are found in the relative rates of molecular evolution (among all gene regions) between sister (putative saltational and non-saltational) lineages.
